# Management of Fournier's gangrene in a newborn: A rare case report and literature review

**DOI:** 10.1016/j.ijscr.2024.109861

**Published:** 2024-06-06

**Authors:** Yufi Aulia Azmi, Dimas Panca Andhika, Johan Renaldo

**Affiliations:** a Department of Urology, Faculty of Medicine, Universitas Airlangga, Dr. Soetomo General Academic Hospital, Surabaya, Indonesia; b Department of Health Sciences, University of Groningen, University Medical Center Groningen, Groningen, the Netherlands; c Departement of Urology, Faculty of Medicine, Universitas Airlangga, Airlangga Academic Hospital, Surabaya, Indonesia

**Keywords:** Fournier's gangrene, Neonates, Case report, Testicular infection

## Abstract

**Introduction:**

Fournier's gangrene (FG) in neonates is less common than in adults, but this case can lead to a poor prognosis. FG is a disease of the genital, perianal, and perineal areas characterized by necrotizing infections. Here, we report a case of a 24-day-old male infant diagnosed with Fournier's gangrene involving the scrotum.

**Case presentation:**

The patient presented with scrotal swelling, fever, erythema, and insect bites on the penile tip that had gradually extended to the proximal area and bilateral scrotum. On physical examination, indurated grayish and blackish-brown scrotal skin with sharp distinction from the surrounding normal skin, erythema, purulence, ulceration, and necrotic tissue were observed. Abdominal X-ray and scrotal ultrasonography revealed gaseous distension of the scrotal region, free fluid on bilateral testes, and enlargement of bilateral testicles. Immediate surgical debridement, along with broad-spectrum antibiotics, was initiated, and a microbiological culture identified the presence of *Pseudomonas aeruginosa.* The patient demonstrated the completed healing of the surgical wound after thirty days of surgical intervention.

**Discussion:**

Fournier's gangrene in neonates is a sporadic case. Our patient presented with multiple predisposing factors, including insect bites and poor hygiene, underscoring the need for heightened clinical suspicion in vulnerable populations. Prompt recognition and intervention are critical, given the rapid progression of FG.

**Conclusion:**

This case underscores the importance of timely diagnosis and early initiation of surgical and medical interventions in neonatal Fournier's gangrene, particularly in cases involving the scrotum.

## Highlights

• 24-day-old infant diagnosed with Fornier gangrene case
• Microbiological culture of the pus swab identified *Pseudomonas aeruginosa.*
• Effective wound care led to excellent healing with minimal scarring

## 1 Introduction

Fournier's gangrene (FG) that occurs in neonates is a rare disease of the genital, perianal, and perineal areas characterized by necrotizing infections. Serious necrotizing infections can follow the fascial plane to involve the abdominal wall as well [1]. The condition was first described by Baurienne in 1764 and later defined by Jean Alfred Fournier in 1883 [2]. FG usually affects adults but is rare in the pediatric population, with 66 % of cases occurring in the first three months of life [3]. Previous research found an annual incidence of 0.8 per million patients, and little is known about the disease in this age group [4]. The incidence of FG in Southern Nigeria shows an average of three cases per year and a high mortality rate (up to 30 %) [5]. FG data on newborns in Indonesia are unavailable, but the mortality rate of FG patients in hospitals in a study population in Indonesia was 26.2 %. In Indonesia, it has previously been identified that the mortality rate due to FG ranges from 17 to 28 % [6].

Fournier gangrene in newborns can occur due to certain risk factors. As with adults, premature and low-birth-weight infants with weakened immune status, as well as infants with poor environmental hygiene, appear to have an increased risk of this rare disease [7]. The variety of presentations makes early diagnosis of NFG difficult [5]. Although rare, Fournier's gangrene remains a disease with severe complications and a high mortality rate. The prognosis is influenced by the timing of medical treatment. This situation possibly leads to septic shock, dysfunction of many organs, and poor prognosis [4]**.**

The process of necrosis generally originates from infection of the anorectum, urogenital tract, or skin of the genitals. Etiological factors reported in the pediatric age group include omphalitis, strangulated hernia, prematurity, diaper rash, varicella infection, circumcision, and perineal skin abscess. Other causes in children include trauma, insect bites, surgery or invasive procedures in the perineal area, urethral instrumentation, burns, and systemic infections. In children, the causative organisms are usually streptococci, staphylococci, and anaerobic. The only prominent feature is poor general hygiene [8]. In this article, we present the case of a 24-day-old baby diagnosed with FG who was referred to our hospital. This report corresponds to the SCARE criteria [9].

## 2 Case presentation

A neonate, 24 days old, was admitted to the hospital due to scrotal swelling that persisted for 11 days. The swelling was preceded by erythema in both scrotal areas, which gradually progressed to a darkened hue. Additionally, the neonate suffered from fever for 11 days before hospitalization. The infant had a previous history of insect bites on the penile tip for 11 days, which eventually extended to the proximal area and bilateral scrotum. The neonate was delivered at full term, weighing 2600 g, and with no recorded episodes of jaundice or cyanosis. No invasive procedures, catheterization, trauma, surgery, steroid administration, prolonged hospitalization, maternal HIV transmission, or other potential predisposing factors were documented.

**Fig. 1:**
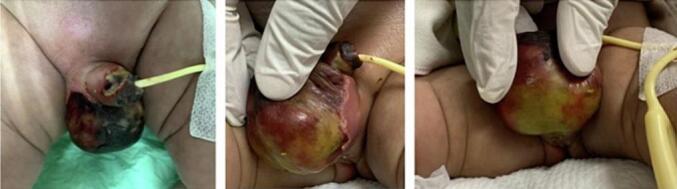
Bilateral scrotal discoloration and swelling with sharp and clear distinction with the normal skin.

**Fig. 2:**
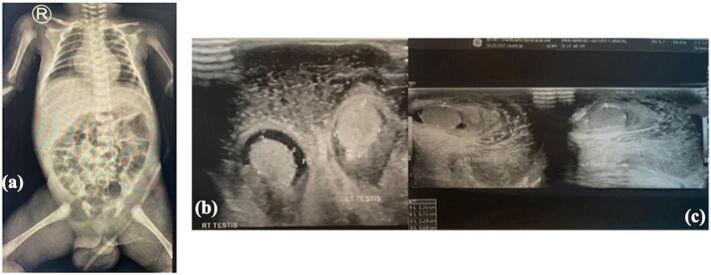
Abdominal X-ray showed a gas appearance in the scrotal region.

**Fig. 3:**
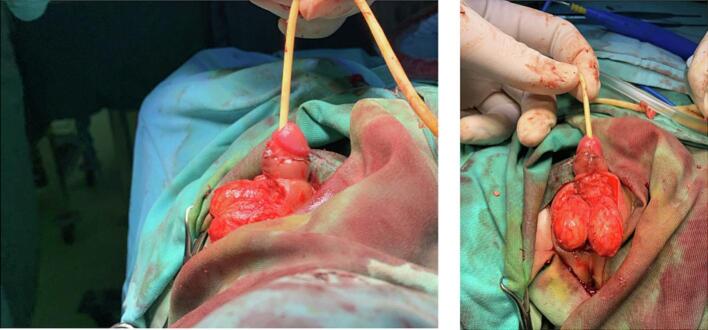
Durante Operation Debridement Necrotomy.

Upon physical examination, the neonate exhibited a healthy and well-nourished appearance with a heart rate of 130 beats per minute, respiratory rate of 38 times per minute, and body temperature of 38.6 °C. Examination of the external genitalia revealed grayish and blackish-brown scrotal skin, indurated with sharp distinction from the surrounding normal skin on both sides (Fig. 1). The dorsal surface of the penis exhibited erythema, purulence, ulceration, and necrotic tissue. The skin of the suprapubic, inguinal, umbilical, and bilateral thighs appeared unaffected. Laboratory investigation revealed a leukocyte count of 34.430/μl, hemoglobin of 9.9 g/dl, platelet count of 239.000/μl, blood urea of 10 mg/dl, creatinine of 0.28 mg/dl, and electrolyte and urine investigation results that were within the normal range. An abdominal X-ray showed gaseous distension of the scrotal region, and on USG, it showed free fluid on bilateral testes and enlargement of bilateral testicles (Fig. 2). Durante operation was done using debridement necrotomy (Fig. 3). In this case, from the ultrasound, the patient's testicles appeared to be enlarged; when necrotomy debridement and testicular exploration were carried out, the size was normal.

In the intraoperative situation, the patient undergoes necrotomy debridement in the necrotic area. Pus is removed maximally. Debridement is carried out until the mucosal surface is reddish. Necrotomy was performed up to 1 cm from the edge of the necrotic area. There is no perianal tunneling. The testicles were found to be intact. The wound was covered with gauze, and scrotal support was performed.

The patient was resuscitated with intravenous fluids, and broad-spectrum antibiotics (Ceftriaxone and Metronidazole) that covered both aerobic and anaerobic organisms were administered immediately upon hospital admission. A catheter was placed, yielding 100 mL/day. By the second day of hospitalization, the patient's symptoms had decreased. Surgical debridement was performed under general anesthesia, with pus samples sent to the microbiology laboratory for bacterial culture. Debridement of the penis and surrounding skin lesions was also carried out in areas of active bleeding. The wound was repeatedly irrigated with normal saline and povidone‑iodine and then packed with an antibiotic tulle dressing and sterile gauze. This dressing was continued throughout the postoperative period. A microbiological culture of the pus swab identified the presence of *Pseudomonas aeruginosa*, and antibiotics were prescribed according to the sensitivity report.

**Fig. 4:**
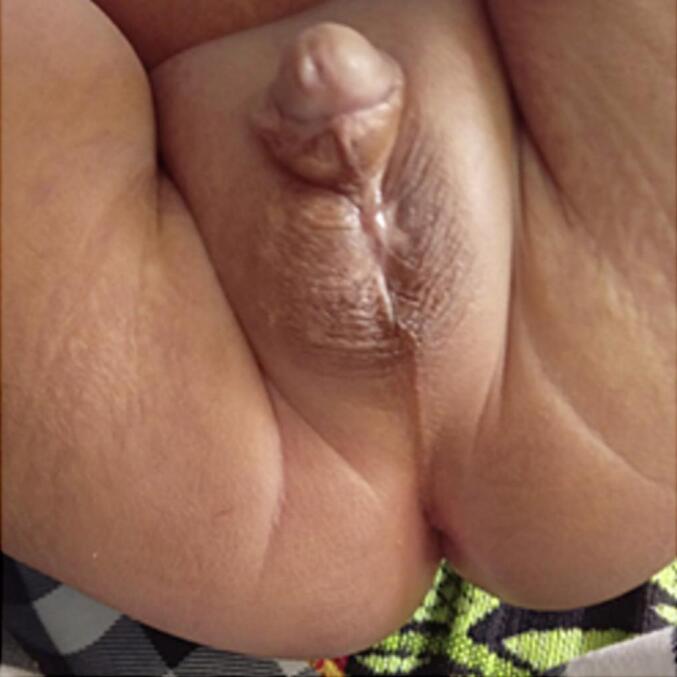
The postoperative surgical wound.

In the post-operative situation, the patient is treated in low care with fluid therapy and empirical broad-spectrum antibiotics according to the bacterial map at the hospital until the pus culture results come out. Routine wound care is carried out twice a day with 0.9 % NaCl. Necrotomy debridement was carried out up to 1 cm from the edge of the necrotic area. In this patient, post-operative care was carried out by regular wound care twice a day. The wound in the scrotum closes secondary without the need for primary scrotal sutures or scrotal flaps. Thirty days after the surgical intervention, the neonate demonstrated excellent overall health status, with completed healing of the surgical wound and minimal scarring (Fig. 4).

## 3 Discussion

The reported incidence of Fournier Gangrene (FG) in the pediatric population is scarce, with cases primarily presenting in the first three months of life [[4], [5], [6], [7], [8]]. As a result, this rare condition must be included in the differential diagnosis when managing acute scrotum. This study presents the case of a 24-day-old infant with FG who presented acute scrotum and leucocytosis.

Our patient presented with several predisposing factors, including insect bites, poor hygiene practices, and delayed medical attention. Our patient did not have a history of prematurity or low birth weight. This shows that FG can be prevented by avoiding risk factors. Other etiological factors reported in the pediatric age group include omphalitis, strangulated hernia, prematurity, diaper rash, varicella infection, circumcision, and perineal skin abscess. Other causes in children include trauma, insect bites, surgery or invasive procedures in the perineal area, urethral instrumentation, burns, and systemic infections [8]. Other research states that certain newborns are more at risk, such as low birth weight, preterm birth, and impaired cellular immunity [7].

Our case's pus swab culture identified *Pseudomonas aeruginosa*, a gram-negative, rod-shaped, flagellated bacterium from the Pseudomonadaceae family. This opportunistic pathogen is obligate aerobic, producing toxins and enzymes that invade the fascial planes, resulting in vascular thrombosis, connective tissue lysis, and eventual gangrene of the overlying skin [10]. While our culture results indicated a single infection, polymicrobial infections can cause extensive and rapidly progressing necrosis, so other microorganisms cannot be entirely ruled out [8]. In our case, inappropriate sample handling, the lack of specific nutrients in the culture medium, or the absence of the medium itself might be the contributing factor to a single infection.

Rapid recognition and diagnosis of FG in newborns is significant because the development of the gangrene process occurs quickly. The clinical picture of the overlying affected skin is variable and may include erythema, edema, cyanosis, bronze discoloration, induration, blistering, and ultimately gangrene [11]. In addition, the presence of a foul odor and crepitation can indicate the presence of anaerobic microorganisms, while the absence of crepitation does not rule out the presence of gas-forming pathogens [12]. In addition, systemic manifestations such as fever, tachycardia, and hypotension may also be observed [2]. In our case, we observed rapid and progressive enlargement of the scrotum and accompanying tissue necrosis. Therefore, clinical suspicion is an important determinant in the timely diagnosis of FG, which can significantly impact patient outcomes.

The laboratory findings in FG cases are less specific but often show an increase in white blood cells, which was also observed in our study [13]. Radiological findings alone cannot confirm the diagnosis of FG, but the presence of edema, inflammation, and gas in the subcutaneous tissues on imaging can aid in supporting the diagnosis [14]. It is noteworthy that soft-tissue air on imaging may be present before clinical crepitus is detected. However, the absence of subcutaneous air in the affected areas does not necessarily rule out the diagnosis of FG [15].

In this case, from the ultrasound, the patient's testicles appeared to be enlarged; when necrotomy debridement and testicular exploration were carried out, the size was normal. This shows that the study needs to be carried out precisely. The afflicted region could extend well beyond the skin's apparent boundaries. The infection may progress to the collarbone in extreme circumstances. The corpora cavernosa, urethra, testicles, and spermatic cord components are typically unaffected unless they are implicated as the initial site of infection [11]. If testicular involvement occurs, this suggests a retroperitoneal or intra-abdominal source of infection [16]. The majority of cases of infection occur due to bacterial infections. Types of bacterial infections include common urinary tract pathogens and pathogens known to cause sexually transmitted diseases. Other possible causes include chemical infections, drugs, and viruses [17].

The management of FG usually involves a comprehensive strategy consisting of the use of broad-spectrum antibiotics and extensive surgical debridement to remove all necrotic tissue up to healthy and viable tissue margins [10]. Fortunately, the testicles and other structures within the tunica vaginalis can generally be preserved from the debridement process [18]. In our case, we immediately initiated a course of broad-spectrum antibiotics, including a combination of ceftriaxone and metronidazole, immediately upon hospital admission. We performed surgical debridement until reaching healthy, viable tissue. This approach successfully prevented the spread of infection to the testes. Adequate wound management facilitated an excellent healing response of the debrided wound, resulting in minimal scarring.

In the treatment of Fournier's gangrene, broad-spectrum antibiotics that cover both aerobic and anaerobic bacteria are recommended. In this case report, treatment was a combination of cephalosporin and metronidazole, and ceftriaxone was chosen over cefotaxime. However, ceftriaxone has a higher risk of severe side effects associated with unconjugated hyperbilirubinemia when used in neonates [19]. The choice of antibiotics in this case was empirically based on the results of the bacterial map in the hospital. Debridement and necrotomy were immediately carried out to take a pus specimen, and a culture was carried out. When the results came out, they were given according to the culture results.

Our study indicates that the presence of risk factors such as a history of insect bites, poor hygiene, and delayed medical care seeking may heighten the susceptibility of infants to FG. Therefore, effective parental health education regarding proper hygiene practices and prompt medical attention can be critical preventive measures against FG morbidity in this population.

## 4 Conclusion

Neonatal FG involving the scrotum is a rare and potentially lethal condition due to sepsis and abdominal involvement. Despite its low incidence, it should be included in the differential diagnosis of acute scrotum, and potential predisposing factors should be identified, even though some cases may be idiopathic. Early diagnosis and prompt surgical intervention are critical, as FG can rapidly extend from the genitalia to adjacent anatomical areas such as the perineum and abdominal wall.

## Financial disclosure

The authors received no financial support for this work.

## Ethical approval

Ethical approval to report this case was obtained from The Hospital Research Ethics Committee, where the patient was admitted.

## Author contributions

Y.A.A.: Conceptualization, Resources, Materials, Methodology, Data collection and/or Processing, Data analysis and/or Interpretation, Writing - original draft.

D.P.A.: Conceptualization, Methodology, Data collection and/or Processing, Supervision, Writing original draft, Review & editing.

J.R.: Supervision, Writing original draft, Data collection and/or Processing, Review & editing.

## Declaration of competing interest

The authors report no declarations of interest.
